# Surface micro- and nano-texturing of stainless steel by femtosecond laser for the control of cell migration

**DOI:** 10.1038/srep36296

**Published:** 2016-11-02

**Authors:** M. Martínez-Calderon, M. Manso-Silván, A. Rodríguez, M. Gómez-Aranzadi, J. P. García-Ruiz, S. M. Olaizola, R. J. Martín-Palma

**Affiliations:** 1 CEIT-IK4 & Tecnun (University of Navarra), Paseo Manuel Lardizábal 15, 20018 San Sebastián, Spain; 2 Departamento de Física Aplicada, Universidad Autónoma de Madrid, Campus de Cantoblanco, 28049 Madrid, Spain; 3 Departamento de Biología Molecular, Universidad Autónoma de Madrid, Campus de Cantoblanco, 28049 Madrid, Spain

## Abstract

The precise control over the interaction between cells and the surface of materials plays a crucial role in optimizing the integration of implanted biomaterials. In this regard, material surface with controlled topographic features at the micro- and nano-scales has been proved to affect the overall cell behavior and therefore the final osseointegration of implants. Within this context, femtosecond (fs) laser micro/nano machining technology was used in this work to modify the surface structure of stainless steel aiming at controlling cell adhesion and migration. The experimental results show that cells tend to attach and preferentially align to the laser-induced nanopatterns oriented in a specific direction. Accordingly, the laser-based fabrication method here described constitutes a simple, clean, and scalable technique which allows a precise control of the surface nano-patterning process and, subsequently, enables the control of cell adhesion, migration, and polarization. Moreover, since our surface-patterning approach does not involve any chemical treatments and is performed in a single step process, it could in principle be applied to most metallic materials.

The detailed study, analysis, and control of the interaction between materials and biological tissues are key issues for the improvement of the overall performance of implants and prostheses integrated into the body. While many polymers have been developed for specific uses given their well-known biocompatibility and corrosion resistance, metallic materials are still required for specific bio-applications in which toughness and durability are of critical importance. Even though Ti-based materials are nowadays well integrated into the body, in particular cases, stainless steel is used for certain prosthetics parts in such fields as prosthetic dentistry, dental implantology, and orthopedics^1^,^2^. Although this particular material possesses very good mechanical properties, it offers a medium-low biocompatibility. Infections and lack of osseointegration are the two principal reasons for implant failure and rejection from the human body^3^. Accordingly, the challenge is to develop a steel surface that interacts with biological tissues in such a way that these problems are overcome.

The specific biological response of the surrounding tissues enormously depends on the surface characteristics of the particular biomaterial^4^,^5^,^6^. Cells respond to synthetic micro- and nano-scale topographic motifs in a wide variety of ways, which depend on factors such as cell type, feature size, geometry, as well as the physico-chemical properties of the bulk substrate material^7^. Therefore, factors such as surface chemistry, topography, energy, and wettability have been proved to play determinant roles in the biomaterial bio-recognition and acceptance by the surrounding tissue^8^,^9^,^10^.

In this regard, the surface properties of biomaterials can be adjusted to the particular needs by means of chemical treatments such as the use of coatings^11^ and plasma surface processes^12^, or by means of physical treatments modifying the surface at the micro- and/or nano-scales. At the micrometer level, the reasoning for this approach is that a rough surface presents a higher developed area than a smooth surface, thus increasing bone anchorage and reinforcing the biomechanical interlocking of the bone with the implant, at least up to a certain level of roughness. At the nanometer level, the particular surface structure has been predicted to control, in addition to the contact angle, such key issues as cell shape and function^13^,^14^. In this line, the roughness can increase the surface energy, thus improving matrix protein adsorption, bone cell migration and proliferation, and, finally, osseointegration^15^.

Among other techniques^16^,^17^, the use of ultrafast lasers to modify the surface topography at the micro- and nano-scales in an extremely controlled manner is considered an excellent option being a very clean and effective technique for this purpose. The main advantage of this technology is probably associated to the fact that the material is much less contaminated since the use of additional materials or gases is avoided^18^. Also, laser-processing is a non-contact procedure which makes it suitable for processing complex shapes^19^. Compared to other type of lasers, with femtosecond (fs) lasers the area affected by heat is minimum^20^ so the mechanical properties of the prosthesis will remain unaltered. Apart from technical advantages, the fact that fs technology is able to precisely machine micro- and nano-features in one single step makes it a very suitable surface modification technique for scaling it up to industrial production.

Several groups have studied the cell behavior in fs-structured surfaces and have obtained very promising results^21^,^22^,^23^. Cunha *et al*.^24^ found that the fs-texturing of titanium with different nanopatterns can induce the stretching of human mesenchymal stem cells (hMSCs) and potentially improve osteoblastic differentiation. Oberringer *et al*.^25^ observed an improvement of endothelial and bone marrow mesenchymal stem cells on fs nano-textured 316L steel, showing that femtosecond surface texturing could improve osseointegration. Dumas *et al*.^26^ proved the ability of fs-structured surfaces to increase osteogenesis and inhibit adipogenesis of MSCs. However, studies regarding the cell migration process through microgrooves and nanostructured surfaces fabricated by fs laser are yet to be conducted, as well as studies on cell morphology and proliferation when exposed to fs laser micro-nano-textured surfaces.

When processing materials with fs-lasers, microstructures are obtained through selective surface ablation of the areas irradiated by the scanning laser beam. In this case, the obtained micro-geometries follow the path defined by the beam. For the generation of nanostructures, the most attractive approach is the fabrication of laser-induced periodic surface structures (LIPSS)^27^,^28^: the variety of LIPSS that can be obtained in metallic surfaces, as well as their periodic nature and homogeneity, makes them an excellent option to study the influence of periodic nanopatterns on cell behavior. For strong absorbing materials, such as most metals, low-spatial-frequency LIPSS (LSFL), also called ripples, are observed with a period generally slightly smaller than the laser wavelength^29^,^30^,^31^. Additionally, it is generally accepted that the irradiation with linearly polarized beams causes LIPSS that are perpendicularly aligned to the incident electric field (E-field) vector^32^,^33^. Therefore, rotating the laser polarization with optical retarders enables the generation of nanopatterns oriented preferentially to a determined specific direction.

Aiming at studying the culturing capabilities of stainless steel-based micropatterns textured at the nanoscale, direct culturing (i.e., with no previous functionalization) of human mesenchymal stem cells (hMSCs) has been carried out. Accordingly, different stainless steel micro- and nano-structured surfaces were fabricated by fs laser taking advantage of the fact that fs LIPSS nanopatterns are generated perpendicular to the laser beam polarization and therefore their orientation can be controlled. Various nanopattern distributions and combinations are evaluated in this work aiming at determining their influence in the cell culture viability, cell migration through the lines, and cell morphology. We expect this work to serve as a generic study which could be exported to most metallic surfaces.

## Methods

### Materials

Commercial AISI304 austenitic stainless steel foils 0.5 mm thick were processed to obtain samples with an area of 100 mm^2^. This material was chosen since it is used for certain prosthesis parts in which good mechanical properties are crucial. Before and after laser processing the samples were cleaned in a 10 min. ultrasonic acetone bath, followed by a 10 min. ethanol bath.

### Micro-nano machining setup

Samples were machined in open air atmosphere with a Ti:Sapphire laser system consisting of a mode-locked oscillator and a regenerative amplifier which was used to generate 130 fs pulses at a central wavelength of 800 nm, with a 1 kHz repetition rate. The pulse energy was adjusted with a two-step setup: a variable attenuator formed by a half-wave plate and a low dispersion polarizer.

The beam diameter (ω_0_) is defined and standardized as the diameter at which the beam irradiance (intensity) has fallen to 1/e^2^ (13.5%) of its peak (ISO 11146–2:2005). In this work, the 8 mm diameter laser beam was focused on the samples using a 10 × microscope objective with a NA of 0.16 to a ω_0_ of approximately 30 μm. This value has been estimated following the analysis of Gaussian beams performed in ref. 34 and based on the characteristics of the femtosecond laser used in this work. It is also important to remark that the actual diameter of a micro-machined line depends on the material’s ablation threshold under the employed experimental conditions and is therefore not always identical to the beam diameter^35^.

**Figure 1:**
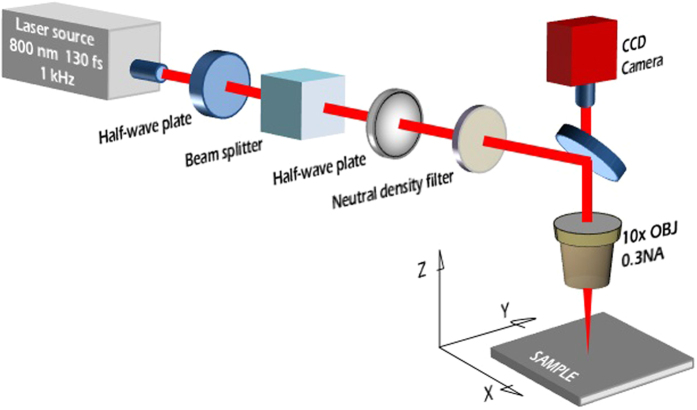
Schematic layout of the femtosecond laser machining setup.

A three-dimensional translational stage was used to move the sample under the laser beam with a velocity of 1 mm/s, thus resulting in 30 pulses per spot. The laser fluence was kept at a value of 2.71 J/cm^2^ throughout all the irradiation process. These laser parameters were chosen based on previous published studies and methods^36^ and were selected because it is known that as a result very homogeneous low-spatial-frequency LIPSS (LSFL) nanopatterns are generated. Finally a CCD camera was used for the online monitoring of the structuring process. The layout of the experimental setup is portrayed in Fig. 1.

**Figure 2:**
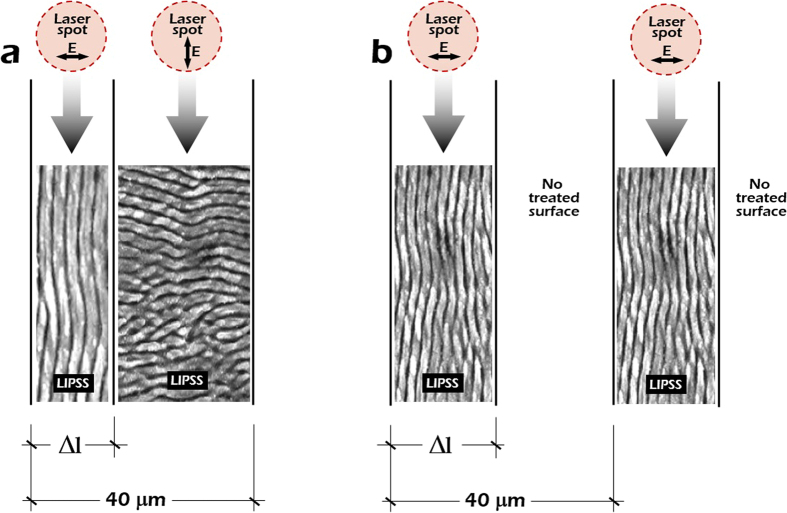
(**a**) Illustration of the surface structure, consisting in alternating microstripes with periodical LIPSS nanopatterns oriented perpendicularly by rotating the laser beam polarization. The total period between alternating stripes is 40 μm. (**b**) Illustration of a similar surface structure alternating vertical LIPSS nanopatterns and non-treated microstripes, again with a period of 40 μm.

The orientation of the LSFL nanopatterns is controlled by rotating the polarization of the laser beam with a half waveplate (λ/2) introduced in the optical pathway. As stated before, LSFL nanopatterns are grown perpendicular to the laser beam polarization. Accordingly, by rotating the polarization vector of the electric field 90° it was possible to obtain LSFL nanopatterns perpendicular to each other. Additionally, different spacing between the lines filled with LSFL nanopatterns was selected by moving the stage. General schemes of the surface structures fabricated on stainless steel are presented in Fig. 2a,b.

### Cell culture

Human bone marrow samples (2–4 ml) from healthy donors were provided by *Hospital Universitario La Princesa* (Madrid, Spain). Cells were collected by centrifugation on 70% Percoll gradient and seeded at 200,000/cm^2^ in Dulbecco’s modified Eagle’s medium with low glucose (DMEM-LG) supplemented with 10% foetal bovine serum (FBS). The medium was replaced twice per week. Before cell culture, the surfaces of the patterns and flat stainless steel controls were exposed to UV light for 10 min, thoroughly washed with phosphate-buffered saline (PBS), placed on a 24-multiwell plate, and seeded with 15,000 cells. Cells were then incubated for 72 h with DMEM-LG adjusted to 10% FBS at 37 °C in 5% CO_2_. After washing twice with PBS, cells were fixed in 3.7% formaldehyde in PBS for 30 min at room temperature (RT). For the immune staining, hMSCs were permeated in 0.5% Triton X-100 in cytoskeleton (CSK) buffer (100 mM NaCl, 10 mM Pipes pH 6.8, 3 mM MgCl2, 3 mM EGTA, and 0.3 M sucrose) for 30 min RT. Samples were blocked with 1% bovine serum albumin in PBS for 1 h at RT. Primary reactions took place with sera from autoimmune mice during 1 h. After washing, the surfaces were incubated in dark conditions for 1 h with Alexa 488 Phalloidin (1:500, Invitrogen) to reveal the Actin cytoskeleton and with 40,6-diamidino-2-phenylindole (1:5000, Calbiochem) to stain the nuclei. After incubation, the surfaces were washed, dehydrated with absolute ethanol, and mounted with Mowiol/Dabco (Calbiochem). Cells were finally visualised in a fluorescence inverted microscope (Olympus IX81, Olympus Corporation, Shinjuku, Tokyo, Japan) coupled to a CCD colour camera. Image analysis of cell density was performed using ImageJ macros over 4 fields of every cell culture condition. All methods carried out in this work accomplish the European guidelines and regulations regarding the work with this type of cells and informed consent was obtained from all subjects. Besides, all experimental protocols were approved by the Ethics Committee of the Hospital Universitario La Princesa and fully complied with the recommendations outlined by the local government.

### Surface characterization

A JPK NanoWizard atomic force microscope (AFM) in the intermittent contact mode using a silicon tip (r < 10 nm; aspect ratio <6:1) with a force constant of 40 N m^−1^ and a resonant frequency of approximately 300 kHz was employed to study the topography of the fabricated stainless steel structures. In addition to this, a 3D field-emission scanning electron microscope (FE-SEM) system supplied by FEI was used to study the topography. Free and open-source software (Gwyddion) was used to perform two dimensional Fast Fourier Transforms (2D-FFT) of the FE-SEM and AFM micro-graphs, which provide an effective way to analyze the LIPSS periods.

## Results and Discussion

### Laser processing

Aiming at studying cell behavior on modified surfaces at the micron- and nano-scales, LIPSS nanopatterns with different orientation were fabricated on stainless steel substrates following the procedure described in the Micro-nano machining setup section. The main objective is assessing the influence of surface morphology on cell growth and migration when cultured on longitudinal and transversal LIPSS nanopatterns. The period between alternating microstripes was fixed (40 μm). Additionally, the width of the microstripes textured at the nanoscale with longitudinal LIPSS, *Δ*_*1*_, was adjusted to be approximately 10 μm given that this value is slightly smaller than the typical diameter of the human mesenchymal stem cells (hMSCs) used for the biological assessment and therefore it could exert a constriction effect.

**Figure 3:**
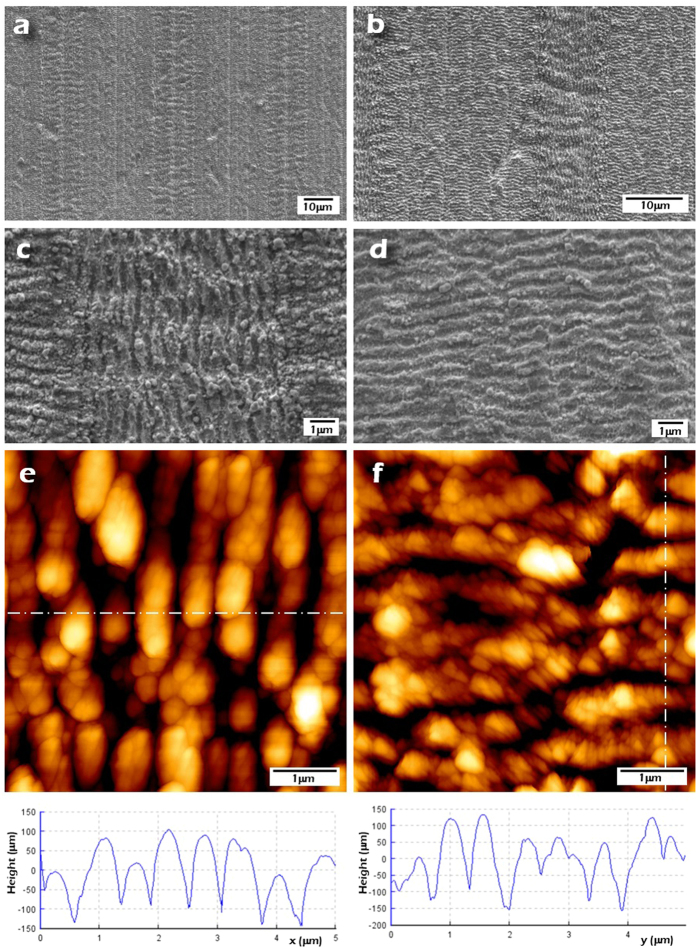
(**a**,**b**) FE-SEM images of the fabricated nanopatterned surfaces with different LIPSS orientations. The width of the stripes filled with longitudinal LIPSS (Δ_1_) is approximately 10 μm. (**c**,**d**) Higher-magnification FE-SEM images of the fabricated LIPSS, longitudinal and transversal. (**e**,**f**) AFM images of the LIPSS nanopatterns and topography profiles that show that the obtained LIPSS period is around 580 nm and that the average height 150 ± 30 nm.

Figure 3a,b show top-view SEM images corresponding to nanopatterned surfaces with different LIPSS orientation which prove that these nanopatterns maintain a good degree of homogeneity. It is also perceived that the 90° polarization rotation has been effectively performed resulting in LIPSS nanopatterns perpendicular to each other. Additionally, Fig. 3c,d show SEM images of higher magnification corresponding to the fabricated nanopatterns with both orientations. From these SEM images it is verified that the fabricated LSFL have a period of 580 nm in average.

The areas with longitudinal and transversal LIPSS were characterized by AFM (Fig. 3e,f). Image analysis determined that the generated nanopatterns show an average height of 150 nm and an acceptable height homogeneity (±30 nm).

**Figure 4:**
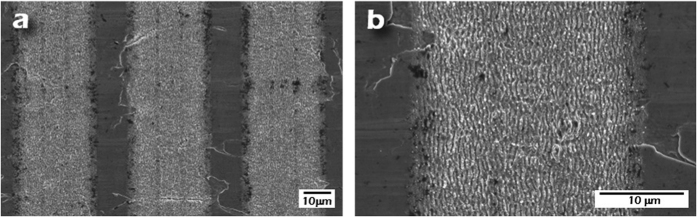
(**a**,**b**) FE-SEM images of the fabricated nanopatterned surfaces with LSFL nanopatterns in the longitudinal direction and separated by no-treated material.

Aiming at determining the specific influence of the transversal LIPSS nanopatterns, another set of samples was fabricated with two clearly differentiated areas combining the two models shown in Fig. 2. The first area was almost equal to the previously described one with perpendicular LIPSS nanopatterns. However, the second area was fabricated following the structure presented in Fig. 2b. It consisted in microstripes filled with longitudinal LIPSS but, instead of separating these lines with transversal LIPSS, a space with non-treated material was allowed. SEM images of this area with the described new patterning are depicted in Fig. 4a,b. The period of the microstripes was kept fixed at 40 μm and the line width (Δ_1_) was 30 μm. The objective in this case was to analyze which area is preferred by the hMSCs to grow and to promote the controlled migration, i.e., zones with LIPSS perpendicular to each other or zones with only longitudinal LIPSS separated by no-treated material.

### Cell adhesion and migration

**Figure 5:**
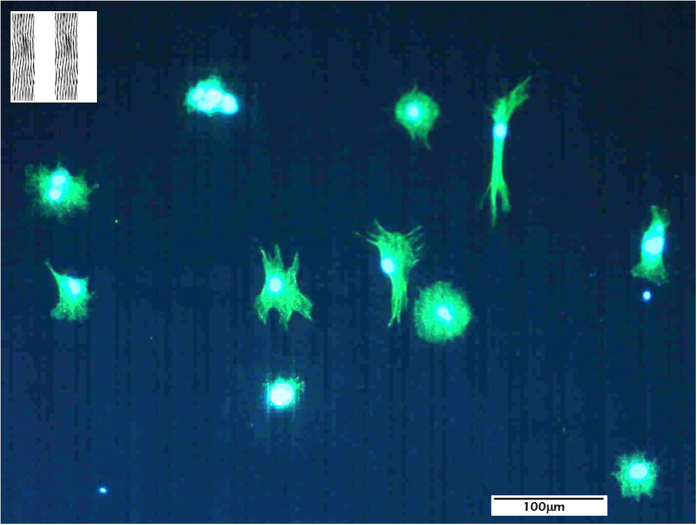
Distribution of hMSCs cultured on LIPSS nanostructured micrometric patterns fabricated on stainless steel, consisting in 10- and 30-μm wide stripes. (inset) top view of the nanostructured micrometric patterns developed for this study.

As a proof of concept, adhesion and migration of human mesenchymal stem cells (hMSCs) on stainless steel-based patterns structured at the micro- and nano-scales was analysed. In the first experiment, hMSCs were cultured on micrometric surface patterns textured at the nanoscale with a total period of 40 μm (inset to Fig. 5). These surface patterns consist in alternating stripes (30 and 10 μm in thickness) in which the LIPSS nanopatterns in the 30-μm wide stripes are oriented parallel to the interfaces. However, in the 10-μm stripes there is no texture at neither the micro- or nano-scale since the original substrate has not been processed.

Figure 5 shows hMSCs distributed over the previously-described LIPSS nanostructured micropatterns. It is evident that cells are not aligned in general. Only in particular cases in which the cells develop filopodia, it is observed that hMSCs tend to present parallel orientation to the linear micropatterns. However, in general terms, it can be affirmed that cells present poorly extended concentric morphologies, which does not allow to infer any particular preferential direction for migration.

**Figure 6:**
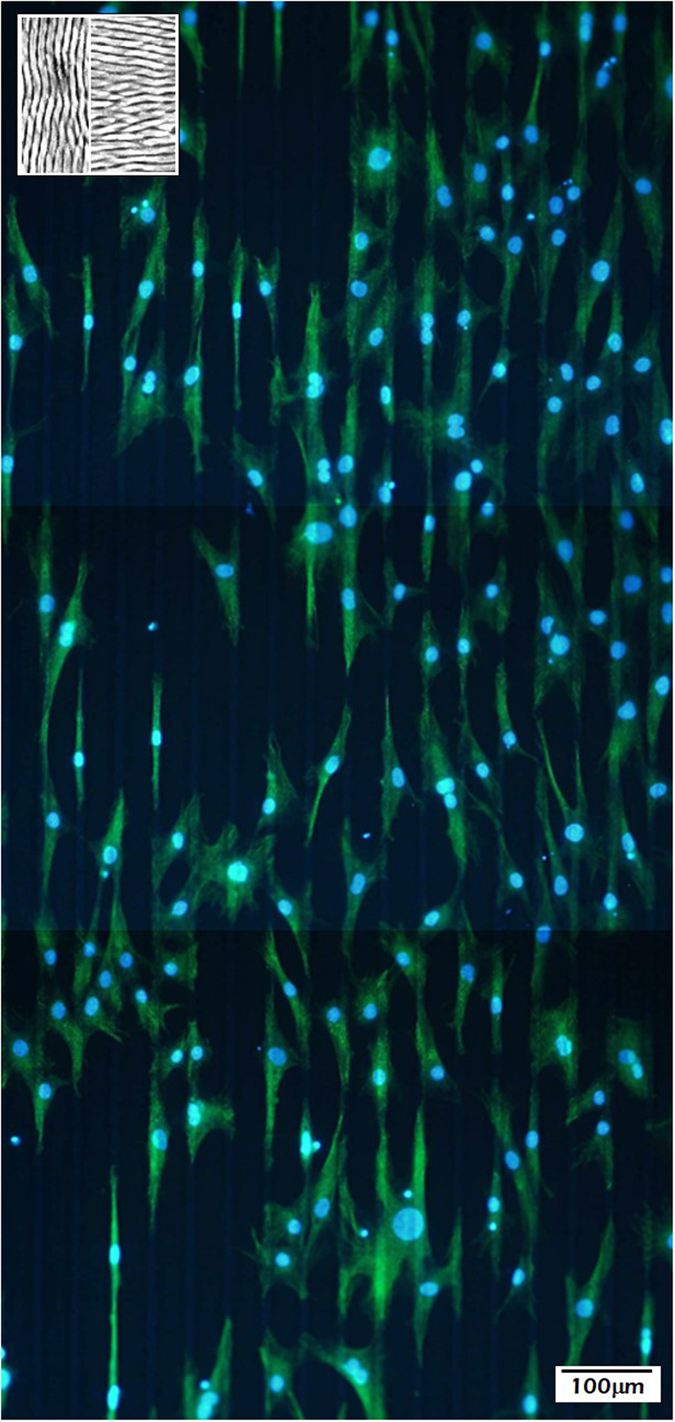
Distribution of hMSCs cultured on nanostructured micrometric patterns. (inset) top view of the LIPSS nanostructured micrometric patterns fabricated on stainless steel, consisting in 10- and 30-μm wide stripes in which the nanopatterns are rotated 90 degrees.

To further asses the influence of the nanostructure on cell behavior, surface patterns with the same micrometric structure, although different nanometric motifs, were fabricated. These surface patterns consist in alternating stripes (with a total period of 40 μm and stripes 10 and 30-μm wide) in which the LIPSS nanopatterns are rotated 90 degrees (inset to Fig. 6). In the 30-μm-widewide stripes, the LIPSS nanopatterns are oriented perpendicular to the stripes, while in the 10-μm stripes the nanopatterns are oriented parallel to the micrometric stripes. It is observed that cells cultured on these structures (Fig. 6) seem to perfectly adapt to the periodicities at the microscale and cells polarize following the micropattern. With respect to their adhesion site, cells restrict dominantly (whenever not strongly influenced by neighboring cells), to the narrower microstripes in which the LIPSS nanopatterns are oriented parallel to the microstripes. Relevantly, as we will see below, the cell density is increased by more than 50% when compared to the previous experiment (comparison performed by the mean of 4 fields) and the small filopodia are no longer observed apart from the dominant polarization microspikes. The asymmetry of the intensity of the actin fibers in some of the more isolated hMSCs further suggests that the cells are migrating along the microchannels following a stretch, bind, and retract process.

**Figure 7:**
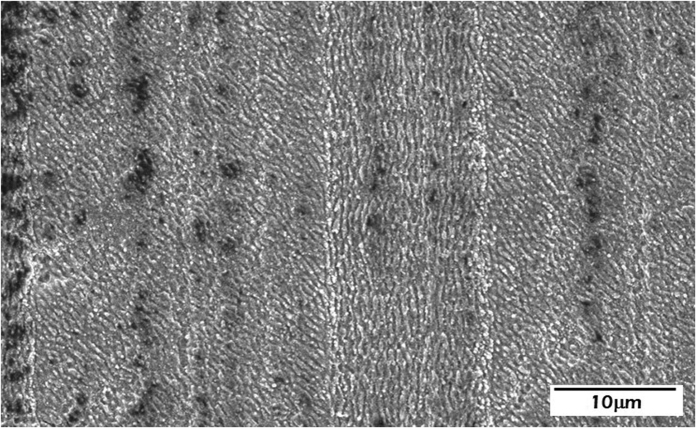
FE-SEM image of the fabricated structure of the micropatterns in zone B consisting in 10-micro-wide stripes filled with longitudinal LIPSS separated by 30-μm-wide stripes with LIPSS rotated by about 40°.

In order to further investigate the role of the LIPPS nanostructured transversal patterns, a different surface pattern was designed, in this case integrating areas with and without transversal periodicities. Again, the surface patterns consisted in alternating stripes (total period 40 μm, and stripes 10 and 30-μm wide). However, in what we termed zone A, the nanopattern is the one presented in the inset to Fig. 5 (i.e., the LIPSS nanopatterns in the 30-μm wide stripes are oriented parallel to them and there is no texture at the nanoscale in the 10-μm stripes). The structure of zone B is portrayed in Fig. 7. Once more, the micrometric stripes are 10 and 30 μm wide. Also, the nanopatterns are oriented parallel to the micrometric stripes in the 10-μm-wide stripes. However, in this case, the LIPSS nanopatterns in the 30-μm-widewide stripes are rotated by about 40 degrees with respect to the microstripes.

**Figure 8:**
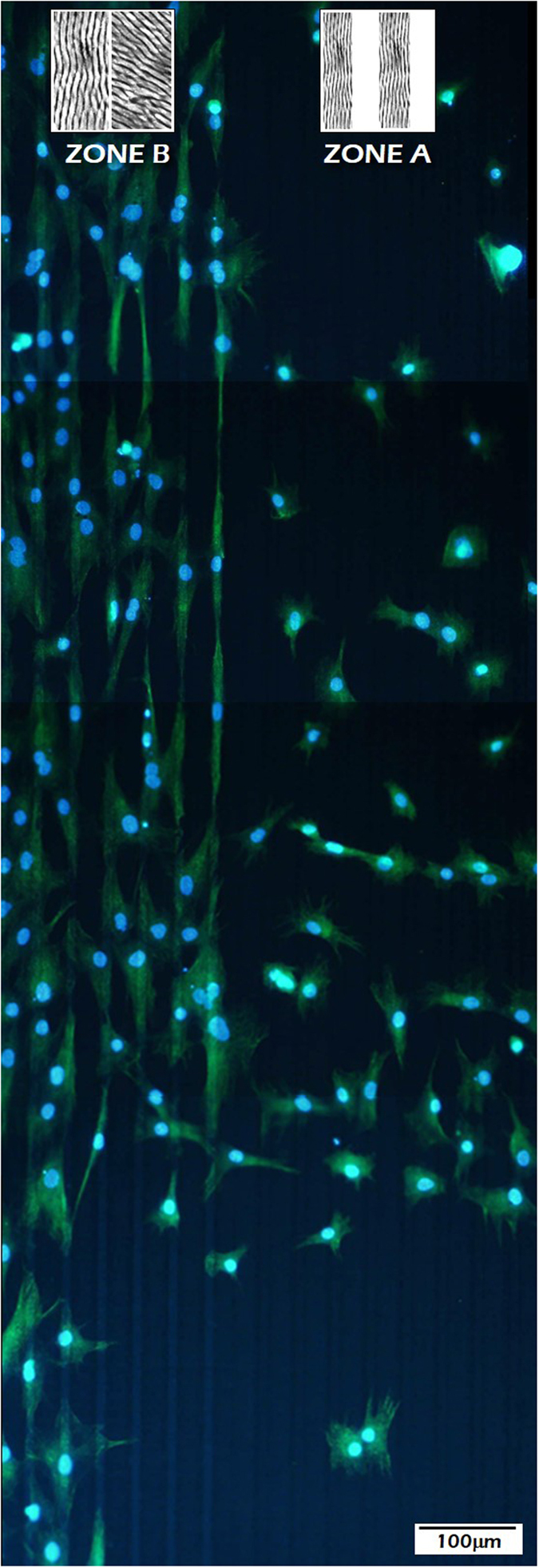
Distribution and migration of hMSCs cultured on the frontier between zones A and B. (insets) top view of the fabricated LIPSS nanostructured micrometric patterns, consisting in 10- and 30-μm wide stripes in which the nanopatterns are rotated 40° (zone B) and stripes filled with longitudinal LIPSS separated by non-treated material (zone A).

Figure 8 shows cell distribution in an area located at the frontier between zones A and B. The experimental results confirm that the absence of transversal LIPSS renders ineffective the guiding influence of the wider longitudinal LIPSS micropattern. However, it can be observed that the promotion of polarization in the stripes with transversal LIPSS induces a call effect to the closer neighboring cells in the area without transversal LIPSS, some of which appear with a polarization normal to that imposed by the underlying micropattern. This result is extremely relevant from the point of view of biocompatibility. If such a call effect on hMSCs could be confirmed by biomolecular analyses, the area could be defined as an osteoinductive structure, in view of the attraction of osteochondral progenitor cells.

The experimental results here presented are partially related to those obtained by D. Brunette *et al*.^37^, which was the first group that reported a directional change in the cell behavior produced by the fabrication of microstructures with different orientation fabricated by photolithography techniques. As discussed in the introduction, very interesting effects can be induced in cell behavior using femtosecond lasers to produce surface changes at the micron- and nano-scales on different materials. However, to our knowledge, no empirical research exists addressing the effect of the orientation and pitch of LIPSS nanopatterns on the overall cell behavior.

Aiming at quantifying cell surface distribution, a morphometric analysis of zones A and B was carried out, based on previous studies related to cell-biomaterial interactions looking at cell-spreading^38^ and cell asymmetry factors^39^. Accordingly, Table 1 shows the experimental results, in which the following parameters were determined: cell density (*d*_*C*_) understood as the number of cells per unit area, cell area (*A*_*C*_) considered as the unitary cell area of adhesion, cell asymmetry (*AS*_*C*_) being a factor between the longest axis of the cell and the shortest axis rotated 90° degrees with respect to the previous, and correlated asymmetry or polarization (*P*_*C*_) calculated by adding to the asymmetry a scalar product between the unitary vector in the direction of the underlying micropattern and the unitary vector in the direction of the longest cell axis.

The data shown in Table 1 verify the results inferred from Fig. 8, i.e., the number of cells per unit area is much larger in zone B than in zone A by a factor over 2. In marked contrast, both cell asymmetry and polarization are much larger in zone A than in zone B. In particular, it should be noted that the value for the polarization in zone B is negligible.

The previous experimental results allow us to conclude that cells preferentially locate on nanopatterns perpendicular to the microstructures, while they tend to avoid unprocessed (flat) stainless steel areas. Also, we attribute this behavior to a purely morphological effect, since FTIR and XPS analysis of processed and unprocessed areas (showed in the supplementary section) did not show a noticeable change in chemical composition.

## Conclusions

Surface patterns with different geometries and distributions were fabricated by femtosecond (fs) laser micro/nano machining technology on stainless steel, aiming at controlling cell adhesion and migration. In particular, it was exploited the fact that the fs Laser-Induced Periodic Surface Structures (LIPSS) formed upon irradiation are defined perpendicular to the laser beam polarization. Therefore their orientation can be precisely controlled. Cell culture experiments with human Mesenchymal Stem Cells (hMSCs) allowed concluding that hMSCs tend to attach and preferentially align to the LIPSS nanopatterns oriented in a longitudinal direction.

These preliminary results allowed us to conclude that it is possible to achieve a very high degree of control over the cell distribution and migration by changing the overall shape of the micropatterns textured at the nanoscale, including their geometry and orientation.

In all, the femtosecond laser-based fabrication method described throughout this work constitutes a simple, clean, and scalable technique which allows a precise control over the surface nano-patterning process. As a result, it enables the control of cell adhesion, migration, and polarization. Moreover, given that our surface-patterning approach does not involve any chemical treatment and is performed in a single step process, it could in principle be applied to most metallic materials.

## Additional Information

**How to cite this article**: Martínez-Calderon, M. *et al*. Surface micro- and nano-texturing of stainless steel by femtosecond laser for the control of cell migration. *Sci. Rep*. **6**, 36296; doi: 10.1038/srep36296 (2016).

**Publisher’s note:** Springer Nature remains neutral with regard to jurisdictional claims in published maps and institutional affiliations.

## Supplementary Material

Supplementary Information

## Figures and Tables

**Table 1:** Quantitative morphometric analysis of hMSCs adhesion onto 10–30 μm wide structures consisting in stripes filled with longitudinal LIPSS separated by non-treated material (zone A) and 10–30 μm wide structures with LIPSS patterns rotated by 40° (zone B).

	*d*_*C*_(cm^−2^)	*A*_*C*_(μm^2^)	*AS*_*C*_	*P*_*C*_
Zone A	(1.1 ± 0.5) × 10^4^	3600 ± 800	7 ± 3	6 ± 3
Zone B	(2.6 ± 0.5) × 10^4^	1900 ± 700	2 ± 1	0 ± 1

Legend: cell density, *d*_*C*_, cell area, *A*_*C*_, cell asymmetry, *AS*_*C*_, correlated asymmetry (Polarization) *P*_*C*_ (see text for definition).
