# Supplementary material for: Surface micro- and nano-texturing of stainless steel by femtosecond laser for the control of cell migration
Source: Sci Rep. 2016 Nov 2;6:36296. doi: 10.1038/srep36296 (PMC5090360; doi:10.1038/srep36296)
Supplement: Supplementary Information [file srep36296-s1.pdf]

Suplementary information

Surface micro- and nano-texturing of stainless steel by femtosecond laser for the control of cell migration

M. Martínez-Calderon[a], Miguel Manso-Silván[b], A. Rodríguez[a], M. Gómez-Aranzadi[a], J.P. García-Ruiz[c], S.M. Olaizola[a], and R.J Martín-Palma[b]

[a] *CEIT-IK4 & Tecnun (University of Navarra), Paseo Manuel Lardizábal 15, 20018 San Sebastián, Spain*

[b] *Departamento de Física Aplicada, Universidad Autónoma de Madrid, Campus de Cantoblanco, 28049 Madrid, Spain*

c Departamento de Biología Molecular, Universidad Autónoma de Madrid, Campus de Cantoblanco, 28049 Madrid, Spain

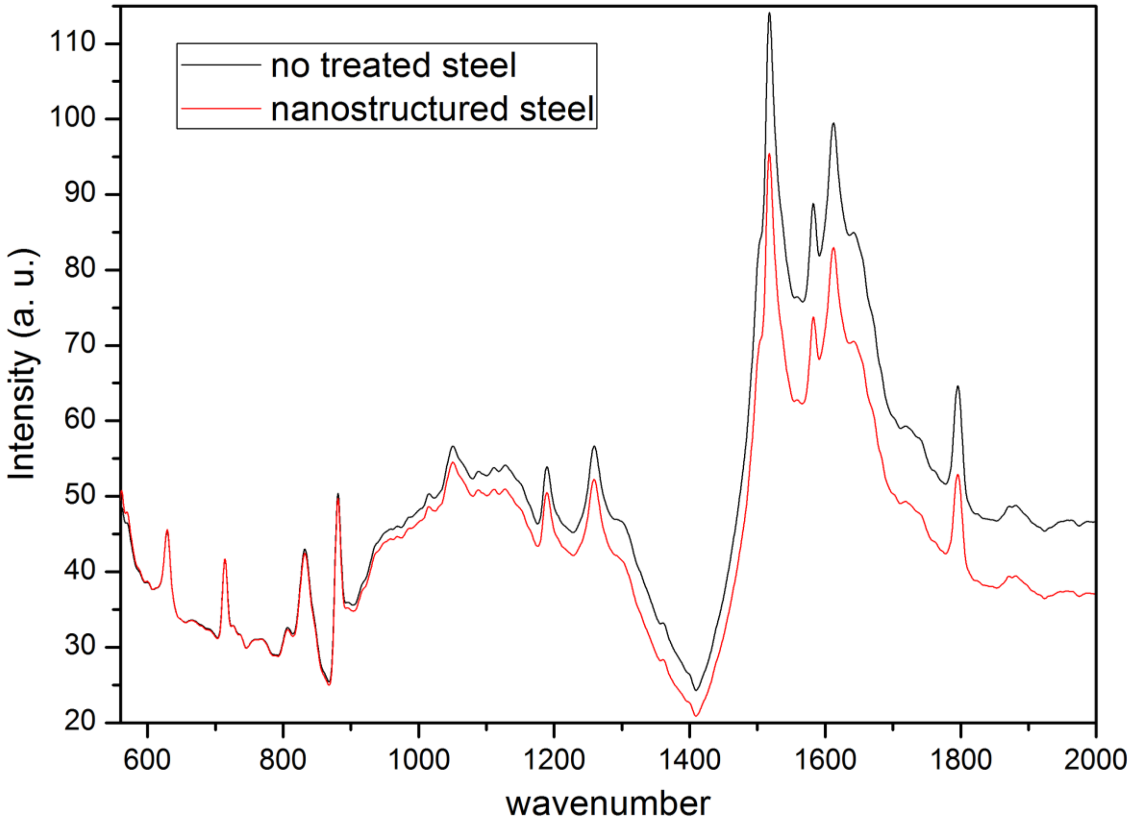

Figure S1. FTIR Spectra corresponding to the no treated areas (black line) and to the nanostructured areas with LIPSS (red line).

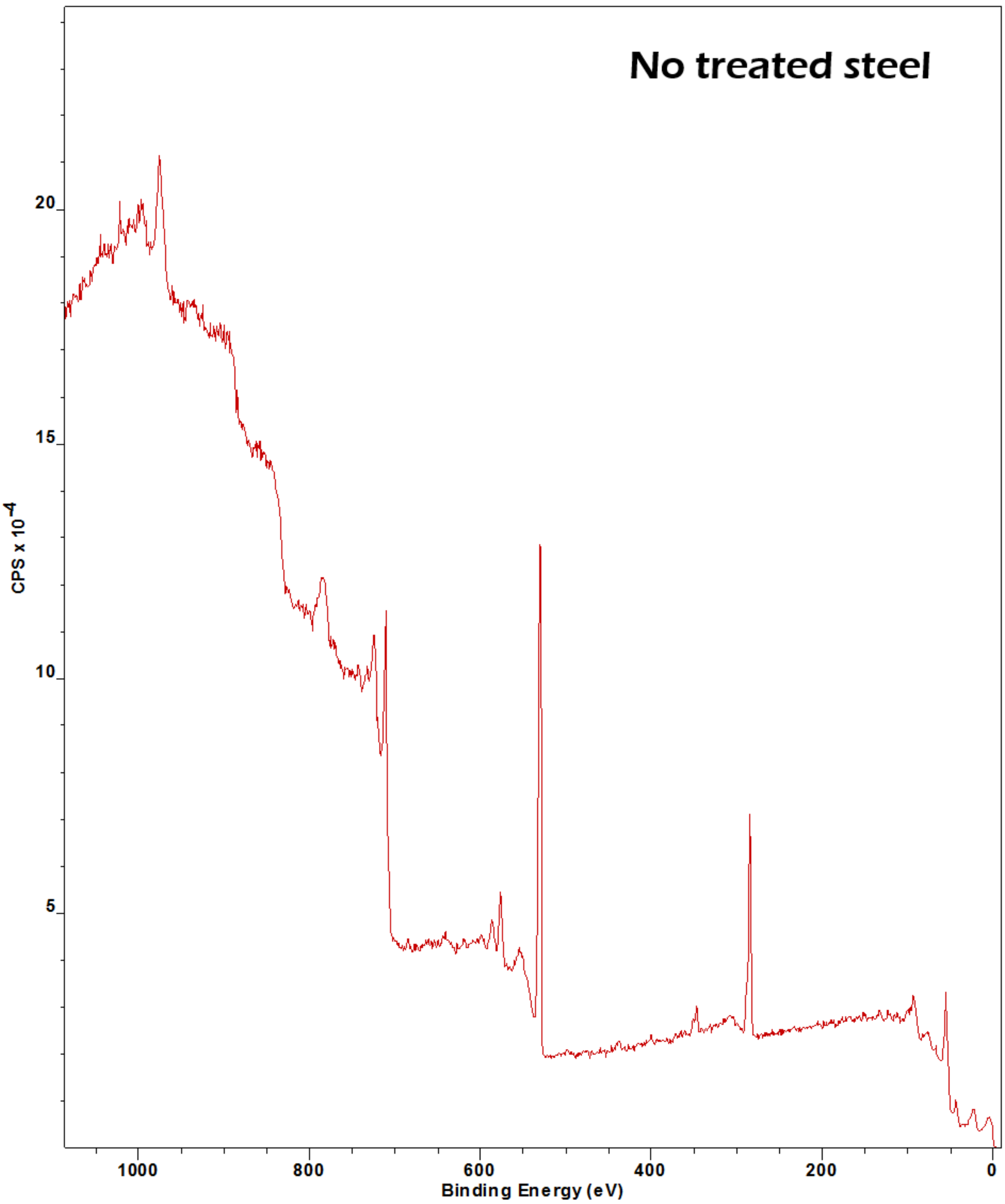

Figure S2. XPS Spectra corresponding to the no treated areas of the steel.

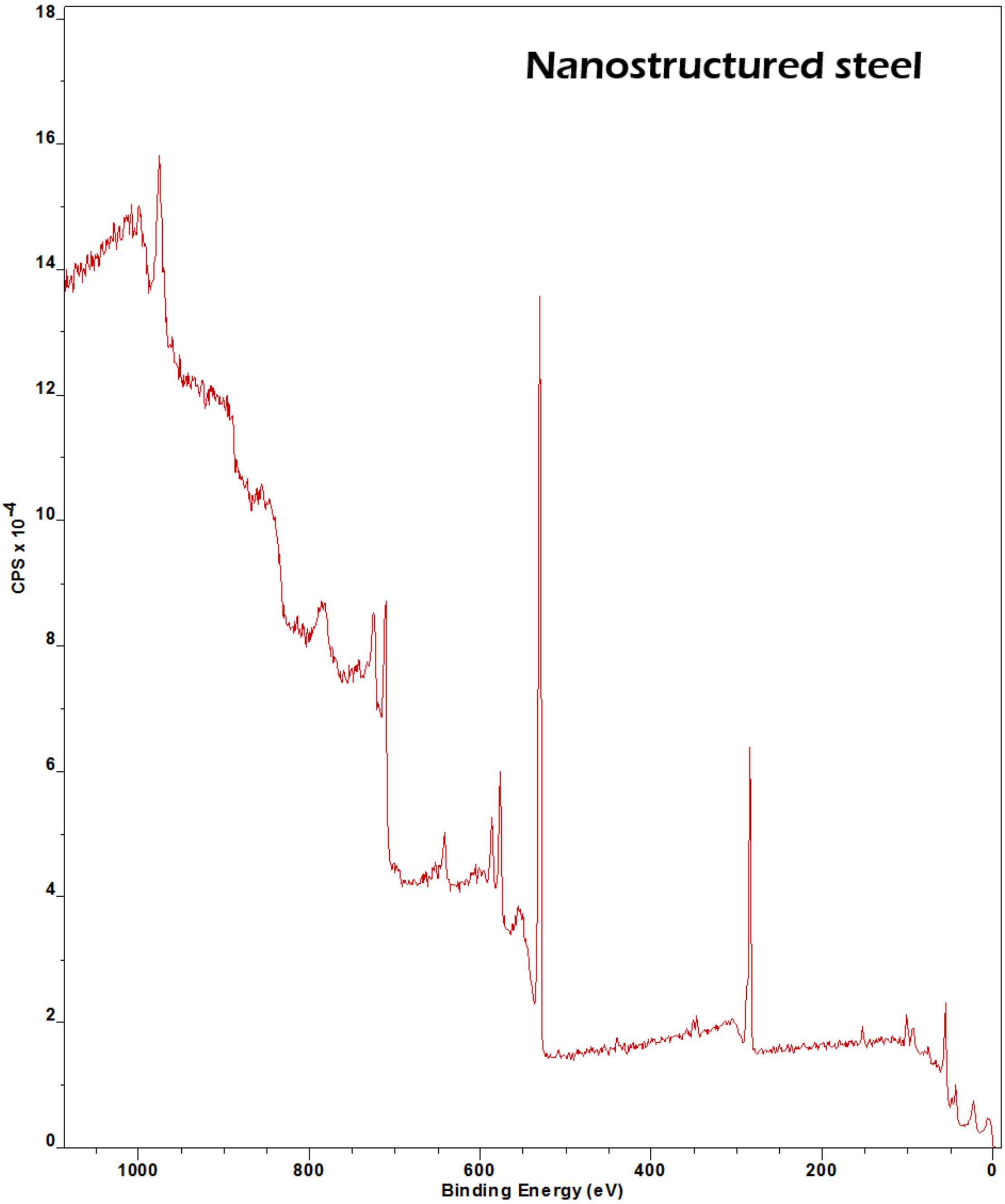

Figure S3. XPS Spectra corresponding to the nanostructured areas of the steel.
